# Exposure to benzylpenicillin after different dosage regimens in growing pigs

**DOI:** 10.1186/s13028-020-00552-0

**Published:** 2020-09-17

**Authors:** Marie Sjölund, Carl Ekstrand, Per Wallgren, Ulf Bondesson, Märit Pringle, Björn Bengtsson

**Affiliations:** 1 Department of Animal Health and Antimicrobial Strategies, National Veterinary Institute, SVA, 751 89 Uppsala, Sweden; 2 Department of Biomedical Sciences and Veterinary Public Health, Faculty of Veterinary Medicine and Animal Husbandry, Swedish University of Agricultural Sciences, Box 7028, 750 07 Uppsala, Sweden; 3 Department of Chemistry, Environment and Feed Hygiene, National Veterinary Institute, SVA, 751 89 Uppsala, Sweden

**Keywords:** *Actinobacillus pleuropneumoniae*, Benzylpenicillin, Dosage regimen, Exposure profile, Pig, Plasma concentration

## Abstract

**Background:**

Penicillin is important for treatment of pigs, but data on its absorption and disposition in pigs are sparse. This is reflected by the variation in recommended dosages in the literature. Inadequate dosage may lead to treatment failure and selection of resistant bacteria. To optimize treatment regimens, plasma exposure to benzylpenicillin for two sustained release formulations of procaine benzylpenicillin for intramuscular administration was studied in growing pigs by means of tandem mass spectrometry (UPLC–MS/MS). One formulation was an aqueous suspension, Ethacilin® vet (ETH), and the other an oily suspension, Ultrapen vet (UPA). Benzylpenicillin exposure after intravenous administration of potassium benzylpenicillin was also explored. Exposure profiles were first studied after single administrations of the approved dosages in healthy pigs and then after repeated administration of different dosages in pigs inoculated intranasally with an *Actinobacillus pleuropneumoniae* serotype 2 strain.

**Results:**

After intravenous administration of benzylpenicillin (n = 6), maximum plasma concentration (*C*_*ma*x_), 1860–9318 µg/L, was observed after 15 min. At four h, plasma concentrations decreased to 15–76 µg/L. After intramuscular administration of ETH (n = 6) *C*_*ma*x_, 1000–4270 µg/L, was observed within one h (*t*_*max*_) in 5 pigs but at four h in one pig. *C*_*ma*x_ for UPA (n = 6), 910–3220 µg/L, was observed within one h in three pigs, but at four or 24 h in three pigs. For both ETH and UPA, the terminal phase was characterized by slow decline compared with intravenous administration. Repeated administration of different dosages of ETH and UPA in pigs inoculated with *A. pleuropneumoniae* (n = 54) showed that the approved dose for UPA (30 mg/kg, qd) but not for ETH (20 mg/kg, qd) gave adequate plasma exposure for bacteria with a penicillin MIC of 500 µg/L. However, more frequent dosing of ETH (bid) or increased dosage gave an adequate exposure.

**Conclusions:**

The approved dosage of ETH provided insufficient plasma exposure for adequate therapy of infections caused by *A. pleuropneumoniae* or other bacteria with a penicillin MIC of 500 µg/L. More frequent ETH dosing (bid) or an increased dosage would improve exposure. The approved dosage of UPA however provided adequate exposure.

## Background

Emergence of antibiotic resistance is driven by the selection pressure exerted by the use of antibiotics. To tackle the increasing burden of resistance, the development of new drugs is crucial [1] and this is also highlighted in the European plan to tackle antibiotic resistance [2]. However, it is recognized that incentives for developing new classes of antibiotics are weak [3]. It is therefore essential to preserve the efficacy of antibiotics already on the market for as long as possible, not the least in veterinary medicine [4, 5]. Prudent and rational use of antibiotics is therefore important not only for the veterinary sector but also in the holistic perspective of “One health” [6]. Prudent use and surveillance to counteract spread of resistant bacteria have made it possible to maintain benzylpenicillin as a first line antibiotic for treatment of the most common infections in production animals such as pigs, cattle and sheep in Sweden [7]. Benzylpenicillin for injection also dominates overall use for pigs in Sweden [8, 9]. Thus, the use of broad-spectrum drugs has largely been avoided in contrast to the situation in many other countries as can be appreciated from data on sales of antibiotics for animals in Europe [10].

Penicillin is a narrow-spectrum antibiotic that was discovered in the late 1920s and has been used in animals for several decades. Although benzylpenicillin dominates antibiotic use for pigs in Sweden, data on basic pharmacokinetic and pharmacodynamic properties (PK/PD) of penicillin in pigs are sparse and emanate from studies performed in healthy animals [11–13]. This is reflected by the variation in recommended dosages in the literature [11–13]. In pigs, mainly slow-release preparations of procaine benzylpenicillin are used for intramuscular injections. The prolonged absorption phase from the injection site of such formulations extends the plasma exposure and consequently the dosing interval. Oil-based formulations of procaine benzylpenicillin are considered to prolong absorption time even further [14].

The antibacterial effect of benzylpenicillin is time dependent and the therapeutic response depends on the time within a dosing interval that the free drug concentration exceeds the minimum inhibitory concentration (MIC) of the infecting bacteria [15]. Thus, to ensure treatment efficacy, knowledge of the relation between plasma exposure and the MIC of pathogenic bacteria is required [5]. For bacteria such as *Actinobacillus pleuropneumoniae*, which may cause severe respiratory disease in pigs, a majority of the wild type isolates have benzylpenicillin MICs of 250 or 500 µg/L [9, 16]. However, the plasma protein binding of benzylpenicillin in pigs is around 45% [17, 18] and to ensure clinical efficacy, the total drug concentration should therefore exceed about 1000 µg/L.

Conclusively, the sparse information on benzylpenicillin exposure after administration of different formulations in pigs warrants further studies of this antibiotic. The objective of this study was therefore to investigate the penicillin exposure in both healthy and experimentally infected growing pigs after administration of benzylpenicillin products available on the Swedish market.

## Methods

### Animals

This study was approved by the regional ethical committee in Uppsala (License C 37/16) and by the Swedish Medical Products Agency (License no. 5.1-2016-16737). All pigs used in the study originated from one specific pathogen-free (SPF) herd [19]. The pigs were either purebred Yorkshire or two- or three-breed hybrids of Yorkshire, Landrace, Hampshire or Duroc. All pigs were 11 to 13 weeks old and weighed between 34 and 49 kg at the beginning of the three trials. None of the pigs received any medication other than the investigated benzylpenicillin formulations preceding or during the trial period.

Part I of the study was conducted as two sub-sets of trials involving nine pigs in their home environment in each trial set. In total, 18 pigs, nine gilts and nine barrows, originating from six litters were used. Each experimental group was balanced for sex and litter of origin. The pigs were kept in their original pens together with their siblings during sampling. The pigs were weighed the day before the trials were conducted.

In Part II of the study, 24 pigs from six litters were used. In Part III, 30 pigs from another six litters were used. In parts II and III, all pigs were transported to the National Veterinary Institute (NVI). On arrival, pigs were weighed and marked for identification. Pigs were allocated to one of four (Part II) or five (Part III) groups with six pigs in each group. The groups were balanced for sex, weight on arrival and litter of origin. Each group was housed in separate rooms with separate ventilation and manure clearing systems previously described in detail [20].

After acclimatization for one week, all pigs in Part II and III were inoculated intranasally with 10^11^ colony forming units of *A. pleuropneumoniae* serotype 2 (strain 700/89, NVI) grown overnight on Bacto PPLO (pleuropneumoniae-like organism) agar at 37°C in a humid atmosphere with 5% CO_2_ and harvested into 0.9% NaCl. Pigs were monitored closely after inoculation and observed for the appearance of respiratory signs. Treatments were initiated when pigs showed signs consistent with an *A. pleuropneumoniae* infection (fever, coughing, increased breathing rate, loss of appetite).

### Dosage regimens

Three different benzylpenicillin formulations were used to study the exposure profiles in healthy pigs and pigs inoculated intranasally with *A. pleuropneumoniae* serotype 2. The three formulations used were: Bensylpenicillin Meda (BPM) containing 148.4–158.1 mg/mL potassium benzylpenicillin (Meda AB, Solna, Sweden); Ethacilin vet (ETH) (Intervet AB, Sollentuna, Sweden) containing 300 mg/mL procaine benzylpenicillin in an aqueous suspension, and Ultrapen vet (UPA) (N-vet, Uppsala, Sweden), containing 300 mg/mL procaine benzylpenicillin in an oily suspension. Dosage regimens for the three parts of the study are shown in Table 1.

**Table 1:** Dosing regimens for three benzylpenicillin formulations used to determine the exposure profiles in grower pigs

Study part	*n* pigs	Medicinal product	Admin route	Loading dose (mg/kg)	Maintenance dose (mg/kg)	Dosing interval	Treatment duration (days)
I	3	Bensylpenicillin Meda	IV	–	10	Single dose	Na*
I	3	Bensylpenicillin Meda	IV	–	20	Single dose	Na*
I	6	Ethacilin vet	IM	–	20	Single dose	Na*
I	6	Ultrapen vet	IM	–	30	Single dose	Na*
II	6	Ethacilin vet	IM	–	20	Every 12 h	3
II	6	Ethacilin vet	IM	–	20	Every 24 h	3
II	6	Ethacilin vet	IM	–	30	Every 24 h	3
II	6	Ultrapen vet	IM	–	30	Every 24 h	3
III	6	Ethacilin vet	IM	–	30	Every 12 h	5
III	6	Ultrapen vet	IM	–	30	Every 24 h	5
III	6	Ultrapen vet	IM	–	30	Every 24 h	3
III	6	Ultrapen vet	IM	60	30	Every 24 h	3
III	6	Ultrapen vet	IM	30^a^	30	Every 24 h	3.5

Table 1. Dosing regimens for three benzylpenicillin formulations used to determine the exposure profiles in healthy pigs and pigs inoculated intranasally with an *Actinobacillus pleuropneumoniae* serotype 2 strain. The three formulations used were: Ethacilin vet (ETH) (Intervet AB, Sollentuna, Sweden) containing 300 mg/ml procaine benzylpenicillin in an aqueous suspension; Ultrapen vet (UPA) (N-vet, Uppsala, Sweden), containing 300 mg/ml procaine benzylpenicillin in an oily suspension, and Bensylpenicillin Meda (BPM) containing potassium benzylpenicillin (Meda AB, Solna, Sweden)

^a^Ethacilin vet 30 mg/kg was administered twelve h before first Ultrapen vet administration

*Not applicable

Intravenous injections (IV) were administered in an ear vein and intramuscular injections (IM) in the lateral neck. All syringes were weighed on a scale with an accuracy of 0.01 g (Tamro Med-Lab AB, Hisings Kärra, Sweden) after they had been filled with the dose calculated and re-weighed after drug-administration to determine the administered amount of benzylpenicillin as exactly as possible.

### Sample collection

In part I, blood samples (n = 9) were collected over 24 h from each pig. In pigs given 10 mg/kg BPM IV, ETH 20 mg/kg IM or UPA 30 mg/kg IM, samples were collected prior to administration (0 h) and at 15, 30, 60, 90, 120, 240 (4 h), 720 (12 h) and 1440 (24 h) min after administration. In pigs given 20 mg/kg BPM IV, samples were collected prior to administration (0 h) and at 15, 30, 60, 120, 240, 480 (8 h), 720 (12 h) and 1440 (24 h) min after administration.

In Part II, blood samples were collected before the first administration (0 h) and at 1, 12 and 24 h after each administration from pigs treated once daily (qd). For pigs treated twice daily (bid), samples were collected before the first administration (0 h) and at 1 and 12 h after each treatment. The last sample in all pigs was collected 24 h after the last administration.

In part III, blood samples were collected before the first administration (0 h) and daily at 1, 12 h and 24 h after each administration. On day two, samples were not collected 12 h after administration due to limitations in the number of samples allowed by the ethics’ consent. For the same reason, samples were not collected 1 h after the second administration on day two from pigs treated bid for 5 days. Moreover, for all pigs treated for 5 days, samples were not collected 1 and 12 h after each administration on day four due to the constraint on the number of sampling occasions allowed. The last sample in all pigs was collected 24 h after the last administration.

Pigs were restrained by the snout to allow for sampling. Blood samples were collected from the jugular vein in 7 mL evacuated plastic tubes (BD Vacutainer^®^, Stockholm, Sweden) containing ethylenediaminetetraacetic acid (EDTA) as additive. Samples were immediately after collection centrifuged for 10 min at 224 *g* (Hettich centrifuge, EBA 200, KRUUSE Svenska AB, Uppsala, Sweden) and the plasma transferred to plastic storage tubes (2 mL Screw Cap Micro Tubes, Sarstedt AB, Helsingborg, Sweden) and stored at − 20 °C for at most 48 h. The samples were then transferred to − 80 °C and stored until analysis.

### Sample analysis

Liquid chromatography tandem mass spectrometry (UPLC–MS/MS) was used for the determination of total benzylpenicillin concentration in plasma. All samples were thawed to room temperature. 200 μL of MilliQ-water and 100 μL of pig plasma were added to each micro tube. 50 μL of MilliQ-water/AcN (1:1) and 50 μL of internal standard solution (2.0 μg of benzylpenicillin-d5 free acid/mL in MilliQ-water: acetonitrile (1:1) were added. The tubes were then Vortexed for 10 min (1650 pulse) after which they were centrifuged at 11500*g* for 5 min. Then 100 μL of sample was transferred to a vial. 400 μL MilliQ-water was added and vortexed for 1 min (1650 pulse). The sample was then injected into the UPLC-MS/MS system Quatro Ultima Pt. The ionization mode used was positive electrospray. The separation was performed with an analytical column: UPLC BEH C18, 1.7 μm 2.1 × 100 mm at 60 ^°^C. The chromatography was performed using a mobile phase consisting of a combination of 0.1% formic acid with water and 0.1% formic acid and acetonitrile in a gradient program with a flow-rate of 200 μL/min. Data acquisition was with Selected Reaction Monitoring (SRM)/Multiple Reaction Monitoring (MRM): penicillin G: precursor m/z 335[M + H] + product m/z 160, penicillin G-d5: precursor m/z 340[M + H]^+^ product m/z 160.

The quantification was carried out with calibration curves constructed by linear regression of the chromatographic peak area ratio (analyte/internal standard) as a function of analyte concentration. The calibration samples were prepared by spiking blank plasma with known amounts of analyte standard. For evaluation of the accuracy, the quantification precision as percentage relative standard deviation (RSD) and the linearity of the method quality control (QC) samples in three different concentrations were prepared by spiking blank plasma. The standards and the QC samples were treated in the same way as the plasma samples obtained from the pigs.

The lower limit of quantification for benzylpenicillin was 10 µg/L in pig plasma and the standard curves were linear in the range 10–15,000 ng/mL of plasma. When a sample had a determined concentration of benzylpenicillin above the highest calibration standard, the plasma sample was diluted in accordance to a standard operation procedure. The relative standard deviation (CV%) of the intra-assay variability for the quality controls used in the analysis of plasma samples containing 10, 30, 752, 7520 and 13,700 ng/mL (*n *= 5 for each concentration) ranged between 0.6% and 9.9%.

## Results

### Single dose exposure to benzylpenicillin after IV and IM dosing to healthy pigs (Part I)

**Fig. 1:**
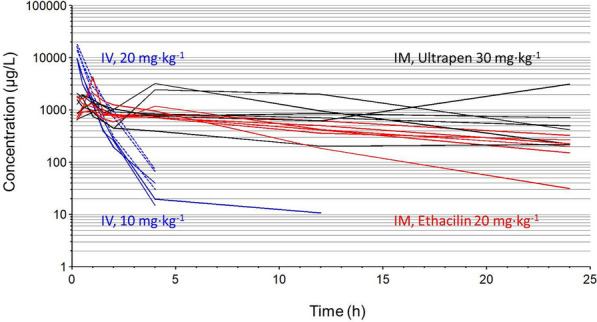
Total plasma concentration–time courses of three benzylpenicillin formulations in healthy grower pigs. Total plasma concentration–time courses of benzylpenicillin in healthy pigs after intravenous administration of either 10 mg/kg (n = 3, solid blue lines) or 20 mg/kg (n = 3, dashed blue lines) and intramuscular administration of 20 mg/kg Ethacilin (n = 6, red solid lines) and 30 mg/kg Ultrapen (n = 6, black solid lines). Each line represents an individual pig

After IV administration of BPM 10 or 20 mg/kg, plasma concentrations decreased from 1860–9318 µg/L at 0.25 h to 15–76 µg/L at 4 h (2–3 orders of magnitude). Individual plasma concentration time courses are shown in Fig. 1. In three of six pigs, a terminal phase with lower elimination rate was observed. After dose-normalization, plasma concentrations obtained after 20 mg/kg superimposed those obtained after 10 mg/kg. Benzylpenicillin plasma concentration was lower than the limit of quantification (10 µg/L) twelve h after administration in four pigs and after 24 h in the remaining two pigs.

Following IM administration, total benzylpenicillin plasma concentrations varied between pigs for both formulations as demonstrated in Fig. 1. After administration of 20 mg/kg ETH, maximum concentrations (*C*_*ma*x_) ranged from 1000 to 4270 µg/L and were observed within one h (*t*_*max*_) in five pigs but was delayed until 4 h in one pig. After administration of 30 mg/kg UPA, *C*_*ma*x_ varied between 910 and 3220 µg/L. In all pigs, a peak in concentration was observed within one h after administration. In three of the pigs this was the *C*_*ma*x_, but in three pigs, there was a second peak at 4 or 24 h which was the *C*_*ma*x_. For both ETH and UPA the terminal phase was characterized by a slow decline compared with IV administration. For both formulations, the plasma concentration was above 100 µg/L in all but one pig, 24 h post administration.

### Exposure to benzylpenicillin after repeated IM dosing to pigs inoculated with *A. pleuropneumoniae* (Part II)

**Fig. 2:**
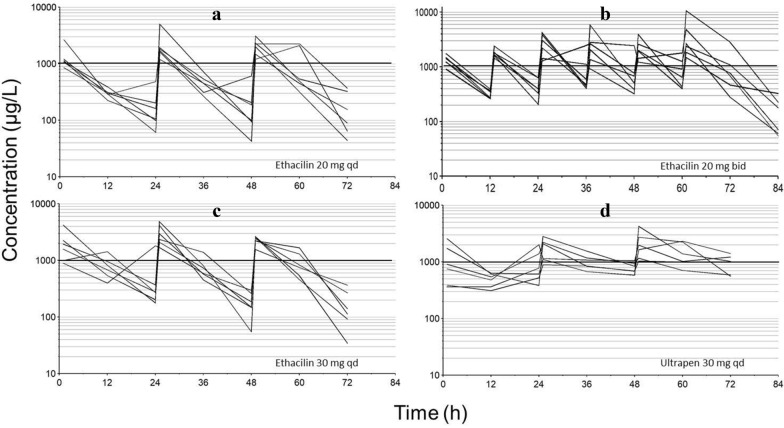
Total plasma concentration–time courses of benzylpenicillin in pigs after repeated intramuscular dosing of different formulations. Total plasma concentration–time courses of benzylpenicillin after repeated intramuscular dosing of different formulations of the drug in pigs experimentally inoculated with *Actinobacillus pleuropneumoniae* serotype 2. Each line represents an individual pig. Plot **a** 20 mg/kg Ethacilin qd to six pigs for 3 days. Plot **b** 20 mg/kg Ethacilin bid to six pigs for 3 days. Plot **c** 30 mg/kg Ethacillin qd to six pigs for 3 days. Plot **d** 30 mg/kg Ultrapen qd to six pigs for 3 days. The horizontal lines denote the approximate total plasma concentration of benzylpenicillin necessary to exceed MIC of 500 µg/L for *A. pleuropneumoniae,* considering a protein binding of 45%

Concentration–time courses of benzylpenicillin in individual pigs after repeated IM dosing of ETH 20 mg/kg qd and bid, ETH 30 mg/kg qd and UPA 30 mg/kg qd for three consecutive days are shown in Fig. 2. Aiming at maintaining a free benzylpenicillin plasma concentration above the MIC for wild type *A. pleuropneumoniae* of 500 µg/L (total concentration ≈ 1000 µg/L) for more than 50% of the dosing-interval, only ETH 20 mg/kg bid and UPA30 mg/kg qd, gave adequate exposures through the last dosing interval (Fig. 2). Administered as ETH 20 mg/kg bid, the total plasma concentration over a dosing-interval was similar to UPA 30 mg/kg qd during the third (48–72 h) day of dosing (Fig. 2). However, there was more pronounced fluctuation with higher peaks and lower trough levels after the ETH bid than the UPA qd regimen. ETH 20 or 30 mg/kg qd did not give adequate exposure.

### Exposure to benzylpenicillin after repeated IM dosing to pigs inoculated with *A. pleuropneumonia* (Part III)

**Fig. 3:**
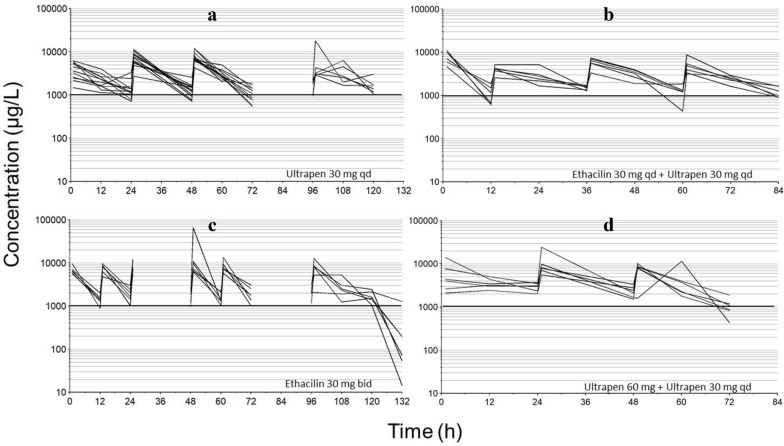
Total plasma concentration–time courses of benzylpenicillin in pigs after repeated intramuscular dosing of different formulations. Total plasma concentration–time courses of benzylpenicillin after repeated intramuscular dosing of different formulations of the drug in pigs experimentally inoculated with *Actinobacillus pleuropneumoniae* serotype 2. Each line represents an individual pig. Discontinuation of the concentration–time courses means that no samples were collected within one dose-interval. Plot **a** 30 mg/kg Ultrapen qd to twelve pigs for 3 days and to six of these for an additional 2 days. Plot **b** A single dose of 30 mg/kg Ethacilin followed by repeated administration of 30 mg/kg Ultrapen qd for 3 days starting at 12 h in six pigs. Plot **c** 30 mg/kg Ethacilin bid for 5 days to six pigs. Plot **d** an initial dose of 60 mg/kg Ultrapen followed by 30 mg/kg qd for 2 days starting at 24 h to six pigs. The horizontal lines denote the approximate total plasma concentration of benzylpenicillin necessary to exceed MIC of 500 µg/L for *A. pleuropneumoniae,* considering a protein binding of 45%

Concentration–time courses of benzylpenicillin after repeated IM dosing of either ETH 30 mg/kg bid for 5 days, UPA 30 mg/kg qd for 5 days, UPA 30 mg/kg qd for 5 days starting 12 h after a start-dose of ETH 30 mg/kg and UPA 30 mg/kg qd for 5 days starting 24 h following a start-dose of UPA 60 mg/kg are shown in Fig. 3. Total benzylpenicillin plasma concentrations were maintained above a MIC of 500 µg/L (total concentration ≈ 1000 µg/L) for all individual pigs and all dosage regimens. The variability within the studied population was similar to that observed in Part II and occurred in all groups studied. This variation appeared larger in animals treated with UPA compared to those treated with ETH. Concentration–time data from all pigs and all doses are provided in supplementary files (Additional file 1).

No adverse reactions following administrations of either of the benzylpenicillin products were observed.

## Discussion

This study shows that the exposure to benzylpenicillin after different dosage-regimens of two depot formulations commonly used in clinical practice in growing pigs is highly variable, irrespective of dosage regimen. Both, the concentration–time course between pigs and between dosing intervals in the same pig varied. The variability appeared more pronounced in pigs administered the oil suspension (UPA), compared to procaine benzylpenicillin administered as an aqueous suspension (ETH). A high variability in benzylpenicillin plasma concentration–time courses after IM administration is in agreement with other studies in pigs [11, 13]. The variability is likely mainly due to the absorption-phase of the benzylpenicillin disposition. However, repeated restraining contributes to a stressful sampling situation in pigs, and stress constitutes a potential source of variability in the pharmacokinetics of drugs [21]. In clinical settings, pigs are not normally subjected to repeated sampling. Hence, the variability in plasma exposure caused by stress would be expected to be less important under field conditions. The observed high variability will however remain a challenge for adequate dose selection in the treatment of bacterial infections in pigs with depot formulations of benzylpenicillin.

The apparently longer terminal half-life after IM administration compared with IV administration indicates absorption rate limited kinetics (flip-flop) where the absorption from the administration site limits the rate of elimination. Double peaks observed in the plasma concentrations of benzylpenicillin in three pigs given the oil suspension (UPA) in Part I of the study, may also be explained by the extended absorption time from the depot formulation. In Parts II and III of the study there were also indications of erratic absorption and delayed C_max_ in some of the pigs given the oil suspension, although double peaks could not be identified due to less frequent sampling than in Part I. The phenomenon of double peaks has previously been reported by Apley and Thacker [11] for intramuscular as well as a needleless air-injection system of 44 mg/kg procaine benzylpenicillin in pigs although information on the suspension medium was not provided. In the current study, the higher dose of the oil suspension, compared to the aqueous suspension, could be a reason for double peaks. However, double peaks were not observed in another study in pigs, in spite of even higher doses (100 mg/kg) of procaine penicillin G after intramuscular injections [13]. Nor was it possible to detect any double peaks after repeated administration of the aqueous suspension in this study. Thus, it seems more likely that the double peaks were a result of the formulation in oil.

The antibacterial effect of β-lactam antibiotics is time-dependent [22]. This means that the time within a dose-interval that the drug exposure exceeds MIC of the target bacteria is decisive for treatment outcome. A clinical point estimate is that the free concentration should exceed MIC of the target bacteria at least 50% of the dose-interval to ensure bactericidal effect [15, 23]. This could be desirable in acute infections caused by pathogens such as *A. pleuropneumoniae*. In vitro studies have shown that if time above MIC increases up to approximately 80% of the dose-interval, the antimicrobial effect increases which could be of clinical importance [22, 24]. The results reported here show that more extended drug exposure over a longer period of time is achieved for the oil suspension of benzylpenicillin, without any observed unwanted effects. This was nicely demonstrated when ETH 30 mg/kg bid was compared with UPA 30 mg/kg qd (Fig. 3). However, all dosage regimens in Part III appeared to produce sufficient exposure to successfully treat an infection caused by bacteria with a MIC of benzylpenicillin less than or equal to 500 µg/L. It should be observed that the total concentration should be twice the MIC for 50% of the dose-interval, considering that the plasma protein binding for benzylpenicillin in pigs is up to 45% [17, 18]. This free fraction is also consistent with what is reported in porcine serum for another penicillin than benzylpenicillin [25]. In contrast to dosage regimens studied in Part III, free plasma concentrations fell below 500 µg/L within 12 h after administration of 20 mg/kg once daily (the approved dose). Consequently, at that dose there is a risk for therapeutic failure when infections are caused by bacteria with a benzylpenicillin MIC of 500 µg/L, e.g. *A. pleuropneumoniae*, of which a majority of the wild type isolates have benzylpenicillin MICs of 250 or 500 µg/L [9, 16]. Hence, the results from this study likely explain the poor therapeutic outcome of 20 mg/kg procaine benzylpenicillin to pigs experimentally infected with *A. pleuropneumoniae* [26–28].

The relatively few numbers of pigs used per dosage regimen may be a limitation to this study, especially in the light of the high variability in plasma exposure. However, using as few animals as possible is well in line with the 3R principle; replace, reduce, refine. A pharmacokinetic model would possibly been able to quantify the variability in experimental data. However, IV-data from two pigs suggest an additional terminal phase with longer half-life than what could be detected in the other pigs. Hence, the area under the curve and subsequently the plasma clearance and the bioavailability would be incorrectly estimated by the model. Since those are two out of three parameters that govern the plasma concentration at steady state, no modelling was performed in this study. Nevertheless, the results clearly indicate how dosage regimens to pigs can be adjusted in clinical practice to achieve successful therapy of bacteria with benzylpenicillin MICs of 500 µg/L. Thus, by applying the information from this study in clinical practice, optimized dosage regimens can be selected for improved treatment outcomes. This is consistent with prudent use guidelines and will improve animal welfare and reduce the risk for emergence of resistance, preserving the effect of antibiotics already on the market.

## Conclusion

The approved IM dosage for ETH (20 mg/kg, qd) provided insufficient plasma benzylpenicillin exposure for therapy of infections caused by *A. pleuropneumoniae* or other bacteria with a wild type MIC of 500 µg/L. More frequent dosing (bid) or an increased dosage would improve exposure. However, the formulation with slower absorption, UPA, provided adequate exposure at the approved IM dosage (30 mg/kg, qd).

## Supplementary information

**Additional file 1:** Plasma concentrations (µg/L) of benzylpenicillin in growing pigs after administration of either an intravenous single dose of Bensylpenicillin Meda (Meda AB, Solna, Sweden) or single or repeated administrations of intramuscular doses of either Ethacilin vet (Intervet AB, Sollentuna, Sweden) and/or Ultrapen vet (N-vet, Uppsala, Sweden).

## Data Availability

All data generated or analyzed during this study are included in this published article [and its Additional file 1].

## Abbreviations

BID: Twice daily
BPM: Bensylpenicillin Meda (Meda AB, Solna, Sweden)
*C*_*max*_: Maximum observed concentration
CV%: Relative standard deviation
EDTA: Ethylenediaminetetraacetic acid
ETH: Ethacilin vet (Intervet AB, Sollentuna, Sweden)
IV: Intravenous
IM: Intramuscular
MIC: Minimum inhibitory concentration
NVI: National Veterinary Institute
PK/PD: Pharmacokinetic and pharmacodynamic properties
PPLO: Pleuropneumoniae-like organism
SPF: Specific pathogen free. Pigs declared free from infections with M*ycoplasma hyopneumoniae, Actinobacillus pleuropneumoniae,* toxigenic *Pasteurella multocida, Brachyspira hyodysenteriae,* swine influenza virus, and *Sarcoptes scabiei* as well as all diseases notifiable to the OIE
UPA: Ultrapen vet (N-vet, Uppsala, Sweden)
UPLC–MS/MS: Liquid chromatography tandem mass spectrometry
*t*_*max*_: Time for *C*_*max*_
QD: Once daily

## References

[1] Bush K, Courvalin P, Dantas G, Davies J, Eisenstein B, Huovinen P (2011). Tackling antibiotic resistance. Nat Rev Microbiol.

[2] European Commission. A European one health action plan against antimicrobial resistance (AMR). 2017.

[3] Tickell S. The antibiotic innovation study: expert voices on a critical need. 2005. http://soapimg.icecube.snowfall.se/strama/AIS%20Report%20React.pdf.

[4] Mouton JW, Ambrose PG, Canton R, Drusano GL, Harbarth S, MacGowan A (2011). Conserving antibiotics for the future: new ways to use old and new drugs from a pharmacokinetic and pharmacodynamic perspective. Drug Resist Updat..

[5] MacGowan A (2011). Revisiting Beta-lactams-PK/PD improves dosing of old antibiotics. Curr Opin Pharmacol.

[6] Kaplan B, Kahn LH, Monath TP (2009). The brewing storm. Vet Ital..

[7] The Swedish Veterinary Association. SVS: Guidelines for the use of antibiotics in production animals-cattle, pigs, sheep and goats. 2017. https://www.svf.se/media/vd5ney4l/svfs-riktlinje-antibiotika-till-produktionsdjur-eng-2017.pdf.

[8] Sjölund M, Backhans A, Greko C, Emanuelson U, Lindberg A (2015). Antimicrobial usage in 60 Swedish farrow-to-finish pig herds. Prev Vet Med.

[9] Swedres-Svarm 2019. Sales of antibiotics and occurrence of resistance in Sweden. Solna/Uppsala; 2020.

[10] European Medicines Agency. Sales of veterinary antimicrobial agents in 31 European countries in 2017. Trends from 2010 to 2017. 9th ESVAC report. 2019.

[11] Apley MD, Thacker B (2003). Determination of the pharmacokinetics of procaine penicillin in swine administered by intra-muscular injection and with a needleless injection device, USA.

[12] Prescott J. Beta-lactam antibiotics: penam penicillins Oxford, Blackwell Publishing Ltd. 121–37. In Antibiotic Therapy in Veterinary medicine. 5th edition. Edited by Giguère S, Prescott JF, Dowling PM. Wiley Blackwell 2013;pp. 135–52.

[13] Ranheim B, Ween H, Egeli AK, Hormazabal V, Yndestad M, Soli NE (2002). Benzathine penicillin G and procaine penicillin G in piglets: comparison of intramuscular and subcutaneous injection. Vet Res Commun.

[14] Summary of Product Characteristics for Ultrapen vet. https://docetp.mpa.se/LMF/Ultrapen%20vet%20300%20mg%20per%20ml%20suspension%20for%20injection%20SmPC_09001bee807a6bf9.pdf.

[15] Drusano GL (2004). Antimicrobial pharmacodynamics: critical interactions of ‘bug and drug’. Nat Rev Microbiol.

[16] Matter D, Rossano A, Limat S, Vorlet-Fawer L, Brodard I, Perreten V (2007). Antimicrobial resistance profile of *Actinobacillus pleuropneumoniae* and *Actinobacillus porcitonsillarum*. Vet Microbiol.

[17] Keen PM (1965). The binding of three penicillins in the plasma of several mammalian species as studied by ultrafiltration at body temperature. Br J Pharmacol Chemother.

[18] Summary of Product Characteristics for Ethacilin vet. https://docetp.mpa.se/LMF/Ethacilin%20vet.%20suspension%20for%20injection%20SmPC_09001bee807a286b.pdf: Swedish Medical Products Agency; 2019.

[19] Wallgren P, Vallgårda J (1993). Serogrisproduktion - presentation, definition och kravlista (SPF pigs–presentation, definition and list of demands). Sv Vet T (Swedish Veterinary J).

[20] Österberg J, Wallgren P (2008). Effects of a challenge dose of *Salmonella* Typhimurium or *Salmonella* Yoruba on the patterns of excretion and antibody responses of pigs. Vet Rec.

[21] Antonia K, Anastasia A, Tesseromatis C (2012). Stress can affect drug pharmacokinetics via serum/tissues protein binding and blood flow rate alterations. Eur J Drug Metab Pharmacokinet.

[22] Frimodt-Moller N, Bentzon MW, Thomsen VF (1986). Experimental infection with *Streptococcus pneumoniae* in mice: correlation of in vitro activity and pharmacokinetic parameters with in vivo effect for 14 cephalosporins. J Infect Dis.

[23] Abdul-Aziz MH, Lipman J, Mouton JW, Hope WW, Roberts JA (2015). Applying pharmacokinetic/pharmacodynamic principles in critically ill patients: optimizing efficacy and reducing resistance development. Semin Respir Crit Care Med.

[24] Craig WA (1998). Pharmacokinetic/pharmacodynamic parameters: rationale for antibacterial dosing of mice and men. Clin Infect Dis.

[25] Teran MT, Sierra M, Garcia JJ, Diez MJ, Arin MJ, Diez MT (1988). Plasma protein binding of an N-pyrrolyl derivative penicillin in several mammalian species. Res Vet Sci.

[26] Sjölund M, de la Fuente AJ, Fossum C, Wallgren P (2009). Responses of pigs to a re-challenge with *Actinobacillus pleuropneumoniae* after being treated with different antimicrobials following their initial exposure. Vet Rec.

[27] Sjölund M, Fossum C, de la Fuente MAJ, Alava M, Juul-Madsen HR, Lampreave F (2011). Effects of different antimicrobial treatments on serum acute phase responses and leucocyte counts in pigs after a primary and a secondary challenge infection with *Actinobacillus pleuropneumoniae*. Vet Rec.

[28] Wallgren P, Segall T, Morner PA, Gunnarsson A (1999). Experimental infections with *Actinobacillus pleuropneumoniae* in pigs-I. Comparison of five different parenteral antibiotic treatments. Zentralbl Veterinarmed B.

