# Supplementary material for: Exposure to benzylpenicillin after different dosage regimens in growing pigs
Source: Acta Vet Scand. 2020 Sep 17;62:55. doi: 10.1186/s13028-020-00552-0 (PMC7499853; doi:10.1186/s13028-020-00552-0)
Supplement: Supplementary file 1 — Additional file 1: Plasma concentrations (µg/L) of benzylpenicillin in growing pigs after administration of either an intravenous single dose of Bensylpenicillin Meda (Meda AB, Solna, Sweden) or single or repeated administrations of intramuscular doses of either Ethacilin vet (Intervet AB, Sollentuna, Sweden) and/or Ultrapen vet (N-vet, Uppsala, Sweden). [file 13028_2020_552_MOESM1_ESM.docx]

**Additional file 1.** Plasma concentrations (µg/L) of benzylpenicillin in growing pigs after administration of either an intravenous single dose of Bensylpenicillin Meda (Meda AB, Solna, Sweden) or single or repeated administrations of intramuscular doses of either Ethacilin vet (Intervet AB, Sollentuna, Sweden) and/or Ultrapen vet (N-vet, Uppsala, Sweden).

| \|  \|  \|  \|  \|  \| **Pig ID** \| \| \| \| \| \| \| --- \| --- \| --- \| --- \| --- \| --- \| --- \| --- \| --- \| --- \| --- \| \| **Study part** \| **Admin route** \| **Medicinal product** \| **Dose**  **(mg/kg)** \| **Time (hours)** \| **1.1** \| **1.2** \| **1.3** \| **1.4** \| **1.5** \| **1.5** \| \| 1 \| IV \| Bensylpenicillin Meda \| 10 \| 0 \| bloq* \| bloq* \| bloq* \| - \| - \| - \| \|  \|  \|  \|  \| 0.25 \| 9555 \| 9202 \| 9318 \| - \| - \| - \| \|  \|  \|  \|  \| 0.5 \| 4302 \| 4525 \| 3113 \| - \| - \| - \| \|  \|  \|  \|  \| 1 \| 1521 \| 1281 \| 1332 \| - \| - \| - \| \|  \|  \|  \|  \| 1.5 \| 640 \| 418 \| 383 \| - \| - \| - \| \|  \|  \|  \|  \| 2 \| 273 \| 195 \| 258 \| - \| - \| - \| \|  \|  \|  \|  \| 4 \| 19.7 \| 40.1 \| 15 \| - \| - \| - \| \|  \|  \|  \|  \| 12 \| 10.7 \| 8.2 \| bloq* \| - \| - \| - \| \|  \|  \|  \|  \| 24 \| bloq* \| bloq* \| bloq* \| - \| - \| - \| \| 1 \| IV \| Bensylpenicillin Meda \| 20 \| 0 \| - \| - \| - \| bloq* \| bloq* \| bloq* \| \|  \|  \|  \|  \| 0.25 \| - \| - \| - \| 18060 \| 16260 \| 13640 \| \|  \|  \|  \|  \| 0.5 \| - \| - \| - \| Missing \| 8990 \| 7560 \| \|  \|  \|  \|  \| 1 \| - \| - \| - \| 4000 \| 2910 \| 2000 \| \|  \|  \|  \|  \| 1.5 \| - \| - \| - \| 737 \| 1000 \| 309 \| \|  \|  \|  \|  \| 2 \| - \| - \| - \| 66.6 \| 76.2 \| 30.2 \| \|  \|  \|  \|  \| 4 \| - \| - \| - \| bloq* \| 8.31 \| bloq* \| \|  \|  \|  \|  \| 12 \| - \| - \| - \| bloq* \| bloq* \| bloq* \| \|  \|  \|  \|  \| 24 \| - \| - \| - \| bloq* \| bloq* \| bloq* \| \|  \|  \|  \|  \|  \|  \|  \|  \|  \|  \|  \| \|  \|  \|  \|  \|  \|  \|  \|  \|  \|  \|  \| \|  \|  \|  \|  \|  \| **1.11** \| **1.12** \| **1.13** \| **1.14** \| **1.15** \| **1.16** \| \| 1 \| IM \| Ethacilin vet \| 20 \| 0 \| bloq* \| bloq* \| bloq* \| bloq* \| bloq* \| bloq* \| \|  \|  \|  \|  \| 0.25 \| 1745 \| 1838 \| 732 \| 652 \| 659 \| 644 \| \|  \|  \|  \|  \| 0.5 \| 1629 \| 1324 \| 1154 \| 902 \| 910 \| 1060 \| \|  \|  \|  \|  \| 1 \| 2143 \| 1137 \| 1339 \| 999 \| 940 \| 4270 \| \|  \|  \|  \|  \| 1.5 \| 1511 \| 1029 \| 828 \| 799 \| 684 \| 901 \| \|  \|  \|  \|  \| 2 \| 1260 \| 731 \| 830 \| 786 \| 594 \| 789 \| \|  \|  \|  \|  \| 4 \| 961 \| 736 \| 712 \| 712 \| 1190 \| 833 \| \|  \|  \|  \|  \| 12 \| 185 \| 416 \| 505 \| 359 \| 424 \| 619 \| \|  \|  \|  \|  \| 24 \| 31.3 \| 151 \| 226 \| 268 \| 201 \| 328 \| \|  \|  \|  \|  \|  \|  \|  \|  \|  \|  \|  \| \|  \|  \|  \|  \|  \|  \|  \|  \|  \|  \|  \| \|  \|  \|  \|  \|  \| **1.21** \| **1.22** \| **1.23** \| **1.24** \| **1.25** \| **1.26** \| \| 1 \| IM \| Ultrapen vet \| 30 \| 0 \| bloq* \| bloq* \| bloq* \| bloq* \| bloq* \| bloq* \| \|  \|  \|  \|  \| 0.25 \| 674 \| 2061 \| 868 \| 1720 \| 1300 \| 1560 \| \|  \|  \|  \|  \| 0.5 \| 734 \| 1682 \| 1046 \| 1970 \| 1890 \| 1150 \| \|  \|  \|  \|  \| 1 \| 910 \| 750 \| 1447 \| 1600 \| 1340 \| 971 \| \|  \|  \|  \|  \| 1.5 \| 614 \| 607 \| 883 \| 1200 \| 1240 \| 1020 \| \|  \|  \|  \|  \| 2 \| 452 \| 444 \| 1035 \| 922 \| 1050 \| 809 \| \|  \|  \|  \|  \| 4 \| 396 \| 2448 \| 3217 \| 839 \| 761 \| 773 \| \|  \|  \|  \|  \| 12 \| 203 \| 2014 \| 960 \| 711 \| 618 \| 859 \| \|  \|  \|  \|  \| 24 \| 218 \| 421 \| 224 \| 495 \| 3120 \| 711 \| \|  \|  \|  \|  \|  \|  \|  \|  \|  \|  \|  \| \|  \|  \|  \|  \|  \|  \|  \|  \|  \|  \|  \| \|  \|  \|  \|  \|  \| **2.1** \| **2.2** \| **2.3** \| **2.4** \| **2.5** \| **2.6** \| \| 2 \| IM \| Ethacilin vet \| 20 \| 0 \| bloq* \| bloq* \| bloq* \| bloq* \| bloq* \| bloq* \| \|  \|  \|  \|  \| 1 \| 1480 \| 1230 \| 1210 \| 908 \| 1740 \| 1420 \| \|  \|  \| Ethacilin vet \| 20 \| 12 \| 275 \| 267 \| 367 \| 264 \| 393 \| 348 \| \|  \|  \|  \|  \| 13 \| 1840 \| 1630 \| 1780 \| 1680 \| 1450 \| 2410 \| \|  \|  \| Ethacilin vet \| 20 \| 24 \| 330 \| 207 \| 633 \| 398 \| 399 \| 635 \| \|  \|  \|  \|  \| 25 \| 4250 \| 1440 \| 1290 \| 2240 \| 3910 \| 3070 \| \|  \|  \| Ethacilin vet \| 20 \| 36 \| 437 \| 1120 \| 2470 \| 473 \| 408 \| 594 \| \|  \|  \|  \|  \| 37 \| 2060 \| 925 \| 2650 \| 5860 \| 1360 \| 2810 \| \|  \|  \| Ethacilin vet \| 20 \| 48 \| 388 \| 321 \| 766 \| 505 \| 682 \| 2450 \| \|  \|  \|  \|  \| 49 \| 1950 \| 3900 \| 2620 \| 1440 \| 2020 \| 1230 \| \|  \|  \| Ethacilin vet \| 20 \| 60 \| 402 \| 433 \| 1250 \| 1790 \| 642 \| 917 \| \|  \|  \|  \|  \| 61 \| 2690 \| 1500 \| 2370 \| 4830 \| 1850 \| 10600 \| \|  \|  \|  \|  \| 72 \| 278 \| 466 \| 1090 \| 689 \| 768 \| 2850 \| \|  \|  \|  \|  \| 84 \| 61 \| 326 \| 179 \| 54.6 \| 72.4 \| 225 \| \|  \|  \|  \|  \|  \|  \|  \|  \|  \|  \|  \| \|  \|  \|  \|  \|  \|  \|  \|  \|  \|  \|  \| \|  \|  \|  \|  \|  \| **2.11** \| **2.12** \| **2.13** \| **2.14** \| **2.15** \| **2.16** \| \| 2 \| IM \| Ethacilin vet \| 20 \| 0 \| bloq* \| bloq* \| bloq* \| bloq* \| bloq* \| bloq* \| \|  \|  \|  \|  \| 1 \| 1200 \| 1090 \| 1130 \| 2640 \| 852 \| 1060 \| \|  \|  \|  \|  \| 12 \| 290 \| 387 \| 224 \| 284 \| 301 \| 312 \| \|  \|  \| Ethacilin vet \| 20 \| 24 \| 61.5 \| 99.5 \| 106 \| 482 \| 161 \| 203 \| \|  \|  \|  \|  \| 25 \| 1630 \| 1720 \| 5030 \| 1920 \| 1190 \| 1810 \| \|  \|  \|  \|  \| 36 \| 264 \| 311 \| 715 \| 448 \| 451 \| 536 \| \|  \|  \| Ethacilin vet \| 20 \| 48 \| 42.5 \| 605 \| 94.1 \| 99.1 \| 206 \| 186 \| \|  \|  \|  \|  \| 49 \| 2000 \| 2310 \| 1210 \| 1470 \| 3080 \| 2240 \| \|  \|  \|  \|  \| 60 \| 319 \| 539 \| 2110 \| 450 \| 494 \| 2260 \| \|  \|  \|  \|  \| 72 \| 43.9 \| 323 \| 64.3 \| 155 \| 89.6 \| 371 \| \|  \|  \|  \|  \|  \|  \|  \|  \|  \|  \|  \| \|  \|  \|  \|  \|  \|  \|  \|  \|  \|  \|  \| \|  \|  \|  \|  \|  \| **2.21** \| **2.22** \| **2.23** \| **2.24** \| **2.25** \| **2.26** \| \| 2 \| IM \| Ethacilin vet \| 30 \| 0 \| bloq* \| bloq* \| bloq* \| bloq* \| bloq* \| bloq* \| \|  \|  \|  \|  \| 1 \| 992 \| 892 \| 1580 \| 2240 \| 4190 \| 754 \| \|  \|  \|  \|  \| 12 \| 1430 \| 399 \| 681 \| 541 \| 822 \| 479 \| \|  \|  \| Ethacilin vet \| 30 \| 24 \| 271 \| 1810 \| 279 \| 202 \| 177 \| 2020 \| \|  \|  \|  \|  \| 25 \| 2090 \| 1690 \| 2970 \| 2350 \| 4890 \| 1150 \| \|  \|  \|  \|  \| 36 \| 735 \| 591 \| 575 \| 1390 \| 805 \| 1080 \| \|  \|  \| Ethacilin vet \| 30 \| 48 \| 148 \| 297 \| 186 \| 259 \| 54.3 \| 1050 \| \|  \|  \|  \|  \| 49 \| 2630 \| 1560 \| 2230 \| 2450 \| 2220 \| 1980 \| \|  \|  \|  \|  \| 60 \| 537 \| 749 \| 1670 \| 834 \| 1300 \| 1020 \| \|  \|  \|  \|  \| 72 \| 34.3 \| 364 \| 113 \| 267 \| 140 \| 1220 \| \|  \|  \|  \|  \|  \|  \|  \|  \|  \|  \|  \| \|  \|  \|  \|  \|  \|  \|  \|  \|  \|  \|  \| \|  \|  \|  \|  \|  \| **2.31** \| **2.32** \| **2.33** \| **2.34** \| **2.35** \| **2.36** \| \| 2 \| IM \| Ultrapen vet \| 30 \| 0 \| bloq* \| bloq* \| bloq* \| bloq* \| bloq* \| bloq* \| \|  \|  \|  \|  \| 1 \| 754 \| 2570 \| 908 \| 388 \| 1730 \| 361 \| \|  \|  \|  \|  \| 12 \| 479 \| 607 \| 543 \| 311 \| 634 \| 365 \| \|  \|  \| Ultrapen vet \| 30 \| 24 \| 2020 \| 385 \| 1390 \| 524 \| 612 \| 774 \| \|  \|  \|  \|  \| 25 \| 1150 \| 2230 \| 2100 \| 889 \| 1110 \| 2810 \| \|  \|  \|  \|  \| 36 \| 1080 \| 1180 \| 837 \| 847 \| 675 \| 1550 \| \|  \|  \| Ultrapen vet \| 30 \| 48 \| 1050 \| 948 \| 694 \| 684 \| 583 \| 838 \| \|  \|  \|  \|  \| 49 \| 1980 \| 1160 \| 1040 \| 1640 \| 4250 \| 2730 \| \|  \|  \|  \|  \| 60 \| 1020 \| 706 \| 994 \| 2320 \| 1390 \| 2250 \| \|  \|  \|  \|  \| 72 \| 1220 \| 587 \| 984 \| 1410 \| 1020 \| 559 \| \|  \|  \|  \|  \|  \|  \|  \|  \|  \|  \|  \| \|  \|  \|  \|  \|  \|  \|  \|  \|  \|  \|  \| \|  \|  \|  \|  \|  \| **3.1** \| **3.2** \| **3.3** \| **3.4** \| **3.5** \| **3.6** \| \| 3 \| IM \| Ethacilin vet \| 30 \| 0 \| bloq* \| bloq* \| bloq* \| bloq* \| bloq* \| bloq* \| \|  \|  \|  \|  \| 1 \| 5530 \| 6200 \| 9510 \| 6580 \| 5720 \| 7070 \| \|  \|  \| Ethacilin vet \| 30 \| 12 \| 1430 \| 1570 \| 896 \| 1380 \| 1290 \| 2020 \| \|  \|  \|  \|  \| 13 \| 6260 \| 9580 \| 8900 \| 8480 \| 4790 \| 7940 \| \|  \|  \| Ethacilin vet \| 30 \| 24 \| 1460 \| 2420 \| 1020 \| 1650 \| 3020 \| 2040 \| \|  \|  \|  \|  \| 25 \| 7680 \| 12000 \| 7530 \| 5460 \| 11800 \| 6750 \| \|  \|  \| Ethacilin vet \| 30 \| 36 \| - \| - \| - \| - \| - \| - \| \|  \|  \| Ethacilin vet \| 30 \| 48 \| 1150 \| 993 \| 1830 \| 1250 \| 1910 \| 2530 \| \|  \|  \|  \|  \| 49 \| 6460 \| 10800 \| 65600 \| 6230 \| 7120 \| 9860 \| \|  \|  \| Ethacilin vet \| 30 \| 60 \| 2160 \| 1484 \| 1310 \| 1320 \| 2120 \| 1040 \| \|  \|  \|  \|  \| 61 \| 5760 \| 13500 \| 7760 \| 9400 \| 7690 \| 7030 \| \|  \|  \| Ethacilin vet \| 30 \| 72 \| 1790 \| 1370 \| 1780 \| 1010 \| 2550 \| 3080 \| \|  \|  \| Ethacilin vet \| 30 \| 84 \| - \| - \| - \| - \| - \| - \| \|  \|  \| Ethacilin vet \| 30 \| 96 \| 1170 \| 2020 \| 2090 \| 1950 \| 3180 \| 2450 \| \|  \|  \|  \|  \| 97 \| 8150 \| 12700 \| Missing \| 5210 \| 8570 \| 8100 \| \|  \|  \| Ethacilin vet \| 30 \| 108 \| 1240 \| 2500 \| 1900 \| 5320 \| 2290 \| 3030 \| \|  \|  \|  \|  \| 120 \| 1520 \| 1610 \| 2140 \| 1040 \| 1410 \| 2440 \| \|  \|  \|  \|  \| 132 \| 197 \| 72.6 \| 1270 \| 14.3 \| 196 \| 53.7 \| \|  \|  \|  \|  \|  \|  \|  \|  \|  \|  \|  \| \|  \|  \|  \|  \|  \|  \|  \|  \|  \|  \|  \| \|  \|  \|  \|  \|  \| **3.11** \| **3.12** \| **3.13** \| **3.14** \| **3.15** \| **3.16** \| \| 3 \| IM \| Ultrapen vet \| 30 \| 0 \| bloq* \| bloq* \| bloq* \| bloq* \| bloq* \| bloq* \| \|  \|  \|  \|  \| 1 \| 5520 \| 5580 \| 6220 \| 6070 \| 4460 \| 2160 \| \|  \|  \|  \|  \| 12 \| 2600 \| 2680 \| 4000 \| 1310 \| 3190 \| 1590 \| \|  \|  \| Ultrapen vet \| 30 \| 24 \| 785 \| 2400 \| 1360 \| 1210 \| 1120 \| 1550 \| \|  \|  \|  \|  \| 25 \| 11300 \| 10400 \| 5720 \| 7550 \| 6150 \| 5470 \| \|  \|  \| Ultrapen vet \| 30 \| 48 \| 1020 \| 931 \| 1510 \| 1670 \| 721 \| 1590 \| \|  \|  \|  \|  \| 49 \| 6790 \| 6340 \| 7410 \| 6480 \| 9460 \| 11900 \| \|  \|  \|  \|  \| 60 \| 2920 \| 4950 \| 3550 \| 2360 \| 2590 \| 3240 \| \|  \|  \| Ultrapen vet \| 30 \| 72 \| 773 \| 1440 \| 1270 \| 768 \| 544 \| 867 \| \|  \|  \| Ultrapen vet \| 30 \| 96 \| 2040 \| 1820 \| 2290 \| 952 \| 2250 \| 1010 \| \|  \|  \|  \|  \| 97 \| 2890 \| 3190 \| 3050 \| 2890 \| 17910 \| 4290 \| \|  \|  \|  \|  \| 108 \| 4520 \| 6330 \| 2560 \| 1680 \| 1990 \| 2740 \| \|  \|  \|  \|  \| 120 \| 1730 \| 1060 \| 1400 \| 1630 \| 3050 \| 1190 \| \|  \|  \|  \|  \|  \|  \|  \|  \|  \|  \|  \| \|  \|  \|  \|  \|  \|  \|  \|  \|  \|  \|  \| \|  \|  \|  \|  \|  \| **3.21** \| **3.22** \| **3.23** \| **3.24** \| **3.25** \| **3.26** \| \| 3 \| IM \| Ultrapen vet \| 30 \| 0 \| bloq* \| bloq* \| bloq* \| bloq* \| bloq* \| bloq* \| \|  \|  \|  \|  \| 1 \| 1480 \| 3250 \| 5210 \| 3580 \| 2440 \| 2580 \| \|  \|  \|  \|  \| 12 \| 1140 \| 1600 \| 2280 \| 2330 \| 1990 \| 1660 \| \|  \|  \| Ultrapen vet \| 30 \| 24 \| 1060 \| 718 \| 1110 \| 1180 \| 1400 \| 3360 \| \|  \|  \|  \|  \| 25 \| 8450 \| 2710 \| 7300 \| 4810 \| 6120 \| 9000 \| \|  \|  \| Ultrapen vet \| 30 \| 48 \| 1210 \| 1510 \| 767 \| 2560 \| 1550 \| 1290 \| \|  \|  \|  \|  \| 49 \| 6630 \| 7060 \| 4310 \| 5980 \| 12000 \| 7780 \| \|  \|  \|  \|  \| 60 \| 2660 \| 2760 \| 2100 \| 3800 \| 2640 \| 2020 \| \|  \|  \|  \|  \| 72 \| 1450 \| 567 \| 1880 \| 1710 \| 1080 \| bloq* \| \|  \|  \|  \|  \|  \|  \|  \|  \|  \|  \|  \| \|  \|  \|  \|  \|  \|  \|  \|  \|  \|  \|  \| \|  \|  \|  \|  \|  \| **3.31** \| **3.32** \| **3.33** \| **3.34** \| **3.35** \| **3.36** \| \| 3 \| IM \| Ultrapen vet \| 60 \| 0 \| bloq* \| bloq* \| bloq* \| bloq* \| bloq* \| bloq* \| \|  \|  \|  \|  \| 1 \| 4390 \| 2620 \| 7650 \| 13900 \| 4020 \| 2100 \| \|  \|  \|  \|  \| 12 \| 3440 \| 3180 \| 5040 \| 4380 \| 3010 \| 2430 \| \|  \|  \| Ultrapen vet \| 30 \| 24 \| 3710 \| 2980 \| 3470 \| 2360 \| 3130 \| 2010 \| \|  \|  \|  \|  \| 25 \| 7060 \| 7910 \| 24100 \| 8210 \| 9810 \| 5530 \| \|  \|  \| Ultrapen vet \| 30 \| 48 \| 1510 \| 3370 \| 2110 \| 2410 \| 1660 \| 2770 \| \|  \|  \|  \|  \| 49 \| 10200 \| 8700 \| 8150 \| 7850 \| 1590 \| 8390 \| \|  \|  \|  \|  \| 60 \| 1790 \| 3970 \| 2280 \| 2200 \| 11500 \| 3730 \| \|  \|  \|  \|  \| 72 \| 824 \| 1900 \| 869 \| 1180 \| 433 \| 1080 \| \|  \|  \|  \|  \|  \|  \|  \|  \|  \|  \|  \| \|  \|  \|  \|  \|  \|  \|  \|  \|  \|  \|  \| \|  \|  \|  \|  \|  \| **3.41** \| **3.42** \| **3.43** \| **3.44** \| **3.45** \| **3.46** \| \| 3 \| IM \| Ethacilin vet \| 30 \| 0 \| bloq* \| bloq* \| bloq* \| bloq* \| bloq* \| bloq* \| \|  \|  \|  \|  \| 1 \| 10100 \| 5980 \| 10000 \| 7010 \| 11100 \| 4600 \| \|  \|  \| Ultrapen vet \| 30 \| 12 \| 670 \| 1890 \| 1170 \| 1500 \| 616 \| 647 \| \|  \|  \|  \|  \| 13 \| 4080 \| 3730 \| 5140 \| 4270 \| 4140 \| 2560 \| \|  \|  \|  \|  \| 24 \| 3030 \| 2410 \| 5210 \| 2790 \| 1680 \| 2180 \| \|  \|  \| Ultrapen vet \| 30 \| 36 \| 1290 \| 1720 \| 1480 \| 1550 \| 1350 \| 1630 \| \|  \|  \|  \|  \| 37 \| 5600 \| 6990 \| 7360 \| 3340 \| 6470 \| 5620 \| \|  \|  \|  \|  \| 48 \| 2630 \| 3850 \| 3940 \| 1890 \| 3500 \| 3260 \| \|  \|  \| Ultrapen vet \| 30 \| 60 \| 1210 \| 1320 \| 1310 \| 1830 \| 1220 \| 436 \| \|  \|  \|  \|  \| 61 \| 5450 \| 3940 \| 4900 \| 3340 \| 8750 \| 3160 \| \|  \|  \|  \|  \| 72 \| 2400 \| 2270 \| 2530 \| 1630 \| 2920 \| 2830 \| \|  \|  \|  \|  \| 84 \| 1000 \| 1660 \| 1270 \| 913 \| 1630 \| 911 \| |
| --- | --- | --- | --- | --- | --- | --- | --- | --- | --- | --- | --- | --- | --- | --- | --- | --- | --- | --- | --- | --- | --- | --- | --- | --- | --- | --- | --- | --- | --- | --- | --- | --- | --- | --- | --- | --- | --- | --- | --- | --- | --- | --- | --- | --- | --- | --- | --- | --- | --- | --- | --- | --- | --- | --- | --- | --- | --- | --- | --- | --- | --- | --- | --- | --- | --- | --- | --- | --- | --- | --- | --- | --- | --- | --- | --- | --- | --- | --- | --- | --- | --- | --- | --- | --- | --- | --- | --- | --- | --- | --- | --- | --- | --- | --- | --- | --- | --- | --- | --- | --- | --- | --- | --- | --- | --- | --- | --- | --- | --- | --- | --- | --- | --- | --- | --- | --- | --- | --- | --- | --- | --- | --- | --- | --- | --- | --- | --- | --- | --- | --- | --- | --- | --- | --- | --- | --- | --- | --- | --- | --- | --- | --- | --- | --- | --- | --- | --- | --- | --- | --- | --- | --- | --- | --- | --- | --- | --- | --- | --- | --- | --- | --- | --- | --- | --- | --- | --- | --- | --- | --- | --- | --- | --- | --- | --- | --- | --- | --- | --- | --- | --- | --- | --- | --- | --- | --- | --- | --- | --- | --- | --- | --- | --- | --- | --- | --- | --- | --- | --- | --- | --- | --- | --- | --- | --- | --- | --- | --- | --- | --- | --- | --- | --- | --- | --- | --- | --- | --- | --- | --- | --- | --- | --- | --- | --- | --- | --- | --- | --- | --- | --- | --- | --- | --- | --- | --- | --- | --- | --- | --- | --- | --- | --- | --- | --- | --- | --- | --- | --- | --- | --- | --- | --- | --- | --- | --- | --- | --- | --- | --- | --- | --- | --- | --- | --- | --- | --- | --- | --- | --- | --- | --- | --- | --- | --- | --- | --- | --- | --- | --- | --- | --- | --- | --- | --- | --- | --- | --- | --- | --- | --- | --- | --- | --- | --- | --- | --- | --- | --- | --- | --- | --- | --- | --- | --- | --- | --- | --- | --- | --- | --- | --- | --- | --- | --- | --- | --- | --- | --- | --- | --- | --- | --- | --- | --- | --- | --- | --- | --- | --- | --- | --- | --- | --- | --- | --- | --- | --- | --- | --- | --- | --- | --- | --- | --- | --- | --- | --- | --- | --- | --- | --- | --- | --- | --- | --- | --- | --- | --- | --- | --- | --- | --- | --- | --- | --- | --- | --- | --- | --- | --- | --- | --- | --- | --- | --- | --- | --- | --- | --- | --- | --- | --- | --- | --- | --- | --- | --- | --- | --- | --- | --- | --- | --- | --- | --- | --- | --- | --- | --- | --- | --- | --- | --- | --- | --- | --- | --- | --- | --- | --- | --- | --- | --- | --- | --- | --- | --- | --- | --- | --- | --- | --- | --- | --- | --- | --- | --- | --- | --- | --- | --- | --- | --- | --- | --- | --- | --- | --- | --- | --- | --- | --- | --- | --- | --- | --- | --- | --- | --- | --- | --- | --- | --- | --- | --- | --- | --- | --- | --- | --- | --- | --- | --- | --- | --- | --- | --- | --- | --- | --- | --- | --- | --- | --- | --- | --- | --- | --- | --- | --- | --- | --- | --- | --- | --- | --- | --- | --- | --- | --- | --- | --- | --- | --- | --- | --- | --- | --- | --- | --- | --- | --- | --- | --- | --- | --- | --- | --- | --- | --- | --- | --- | --- | --- | --- | --- | --- | --- | --- | --- | --- | --- | --- | --- | --- | --- | --- | --- | --- | --- | --- | --- | --- | --- | --- | --- | --- | --- | --- | --- | --- | --- | --- | --- | --- | --- | --- | --- | --- | --- | --- | --- | --- | --- | --- | --- | --- | --- | --- | --- | --- | --- | --- | --- | --- | --- | --- | --- | --- | --- | --- | --- | --- | --- | --- | --- | --- | --- | --- | --- | --- | --- | --- | --- | --- | --- | --- | --- | --- | --- | --- | --- | --- | --- | --- | --- | --- | --- | --- | --- | --- | --- | --- | --- | --- | --- | --- | --- | --- | --- | --- | --- | --- | --- | --- | --- | --- | --- | --- | --- | --- | --- | --- | --- | --- | --- | --- | --- | --- | --- | --- | --- | --- | --- | --- | --- | --- | --- | --- | --- | --- | --- | --- | --- | --- | --- | --- | --- | --- | --- | --- | --- | --- | --- | --- | --- | --- | --- | --- | --- | --- | --- | --- | --- | --- | --- | --- | --- | --- | --- | --- | --- | --- | --- | --- | --- | --- | --- | --- | --- | --- | --- | --- | --- | --- | --- | --- | --- | --- | --- | --- | --- | --- | --- | --- | --- | --- | --- | --- | --- | --- | --- | --- | --- | --- | --- | --- | --- | --- | --- | --- | --- | --- | --- | --- | --- | --- | --- | --- | --- | --- | --- | --- | --- | --- | --- | --- | --- | --- | --- | --- | --- | --- | --- | --- | --- | --- | --- | --- | --- | --- | --- | --- | --- | --- | --- | --- | --- | --- | --- | --- | --- | --- | --- | --- | --- | --- | --- | --- | --- | --- | --- | --- | --- | --- | --- | --- | --- | --- | --- | --- | --- | --- | --- | --- | --- | --- | --- | --- | --- | --- | --- | --- | --- | --- | --- | --- | --- | --- | --- | --- | --- | --- | --- | --- | --- | --- | --- | --- | --- | --- | --- | --- | --- | --- | --- | --- | --- | --- | --- | --- | --- | --- | --- | --- | --- | --- | --- | --- | --- | --- | --- | --- | --- | --- | --- | --- | --- | --- | --- | --- | --- | --- | --- | --- | --- | --- | --- | --- | --- | --- | --- | --- | --- | --- | --- | --- | --- | --- | --- | --- | --- | --- | --- | --- | --- | --- | --- | --- | --- | --- | --- | --- | --- | --- | --- | --- | --- | --- | --- | --- | --- | --- | --- | --- | --- | --- | --- | --- | --- | --- | --- | --- | --- | --- | --- | --- | --- | --- | --- | --- | --- | --- | --- | --- | --- | --- | --- | --- | --- | --- | --- | --- | --- | --- | --- | --- | --- | --- | --- | --- | --- | --- | --- | --- | --- | --- | --- | --- | --- | --- | --- | --- | --- | --- | --- | --- | --- | --- | --- | --- | --- | --- | --- | --- | --- | --- | --- | --- | --- | --- | --- | --- | --- | --- | --- | --- | --- | --- | --- | --- | --- | --- | --- | --- | --- | --- | --- | --- | --- | --- | --- | --- | --- | --- | --- | --- | --- | --- | --- | --- | --- | --- | --- | --- | --- | --- | --- | --- | --- | --- | --- | --- | --- | --- | --- | --- | --- | --- | --- | --- | --- | --- | --- | --- | --- | --- | --- | --- | --- | --- | --- | --- | --- | --- | --- | --- | --- | --- | --- | --- | --- | --- | --- | --- | --- | --- | --- | --- | --- | --- | --- | --- | --- | --- | --- | --- | --- | --- | --- | --- | --- | --- | --- | --- | --- | --- | --- | --- | --- | --- | --- | --- | --- | --- | --- | --- | --- | --- | --- | --- | --- | --- | --- | --- | --- | --- | --- | --- | --- | --- | --- | --- | --- | --- | --- | --- | --- | --- | --- | --- | --- | --- | --- | --- | --- | --- | --- | --- | --- | --- | --- | --- | --- | --- | --- | --- | --- | --- | --- | --- | --- | --- | --- | --- | --- | --- | --- | --- | --- | --- | --- | --- | --- | --- | --- | --- | --- | --- | --- | --- | --- | --- | --- | --- | --- | --- | --- | --- | --- | --- | --- | --- | --- | --- | --- | --- | --- | --- | --- | --- | --- | --- | --- | --- | --- | --- | --- | --- | --- | --- | --- | --- | --- | --- | --- | --- | --- | --- | --- | --- | --- | --- | --- | --- | --- | --- | --- | --- | --- | --- | --- | --- | --- | --- | --- | --- | --- | --- | --- | --- | --- | --- | --- | --- | --- | --- | --- | --- | --- | --- | --- | --- | --- | --- | --- | --- | --- | --- | --- | --- | --- | --- | --- | --- | --- | --- | --- | --- | --- | --- | --- | --- | --- | --- | --- | --- | --- | --- | --- | --- | --- | --- | --- | --- | --- | --- | --- | --- | --- | --- | --- | --- | --- | --- | --- | --- | --- | --- | --- | --- | --- | --- | --- | --- | --- | --- | --- | --- | --- | --- | --- | --- | --- | --- | --- | --- | --- | --- | --- | --- | --- | --- | --- | --- | --- | --- | --- | --- | --- | --- | --- | --- | --- | --- | --- | --- | --- | --- | --- | --- | --- | --- | --- | --- | --- | --- | --- | --- | --- | --- | --- | --- | --- | --- | --- | --- | --- | --- | --- | --- | --- | --- | --- | --- | --- | --- | --- | --- | --- | --- | --- | --- | --- | --- | --- | --- | --- | --- | --- | --- | --- | --- | --- | --- | --- | --- | --- | --- | --- | --- | --- | --- | --- | --- | --- | --- | --- | --- | --- | --- | --- | --- | --- | --- | --- | --- | --- | --- | --- | --- | --- | --- | --- | --- | --- | --- | --- | --- | --- | --- | --- | --- | --- | --- | --- | --- | --- | --- | --- | --- | --- | --- | --- | --- | --- | --- | --- | --- | --- | --- | --- | --- | --- | --- | --- | --- | --- | --- | --- | --- | --- | --- | --- | --- | --- | --- | --- | --- | --- | --- | --- | --- | --- | --- | --- | --- | --- | --- | --- | --- | --- | --- | --- | --- | --- | --- | --- | --- | --- | --- | --- | --- | --- | --- | --- | --- | --- | --- | --- | --- | --- | --- | --- | --- | --- | --- | --- | --- | --- | --- | --- | --- | --- | --- | --- | --- | --- | --- | --- | --- | --- | --- | --- | --- | --- | --- | --- | --- | --- | --- | --- | --- | --- | --- | --- | --- | --- | --- | --- | --- | --- | --- | --- | --- | --- | --- | --- | --- | --- | --- | --- | --- | --- | --- | --- | --- | --- | --- | --- | --- | --- | --- | --- | --- | --- | --- | --- | --- | --- | --- | --- | --- | --- | --- | --- | --- | --- | --- | --- | --- | --- | --- | --- | --- | --- | --- | --- | --- | --- | --- | --- | --- | --- | --- | --- | --- | --- | --- | --- | --- | --- | --- | --- | --- | --- | --- | --- | --- | --- | --- | --- | --- | --- | --- | --- | --- | --- | --- | --- | --- | --- | --- | --- | --- | --- | --- | --- | --- | --- | --- | --- | --- | --- | --- | --- | --- | --- | --- | --- | --- | --- | --- | --- | --- | --- | --- | --- | --- | --- | --- | --- | --- | --- | --- | --- | --- | --- | --- | --- | --- | --- | --- | --- | --- | --- | --- | --- | --- | --- | --- | --- | --- | --- | --- | --- | --- | --- | --- | --- | --- | --- | --- | --- | --- | --- | --- | --- | --- | --- | --- | --- | --- | --- | --- | --- | --- | --- | --- | --- | --- | --- | --- | --- | --- | --- | --- | --- | --- | --- | --- | --- | --- | --- | --- | --- | --- | --- | --- | --- | --- | --- | --- | --- | --- | --- | --- | --- | --- | --- | --- | --- | --- | --- | --- | --- | --- | --- | --- | --- | --- | --- | --- | --- | --- | --- | --- | --- | --- | --- | --- | --- | --- | --- | --- | --- | --- | --- | --- | --- | --- | --- | --- | --- | --- | --- | --- | --- | --- | --- | --- | --- | --- | --- | --- | --- | --- | --- | --- | --- | --- | --- | --- | --- | --- | --- | --- | --- | --- | --- | --- | --- | --- | --- | --- | --- | --- | --- | --- | --- | --- | --- | --- | --- | --- | --- | --- | --- | --- | --- | --- | --- | --- | --- | --- | --- | --- | --- | --- | --- | --- | --- | --- | --- | --- | --- | --- | --- | --- | --- | --- | --- | --- | --- | --- | --- | --- | --- | --- | --- | --- | --- | --- | --- | --- | --- | --- | --- | --- | --- | --- | --- | --- | --- | --- | --- | --- | --- | --- | --- | --- | --- | --- | --- | --- | --- | --- | --- | --- | --- | --- | --- | --- | --- | --- | --- | --- | --- | --- | --- | --- | --- | --- | --- | --- | --- | --- | --- | --- | --- | --- | --- | --- | --- | --- | --- | --- | --- | --- | --- | --- | --- | --- | --- | --- | --- | --- | --- | --- | --- | --- | --- | --- | --- | --- | --- | --- | --- | --- | --- | --- | --- | --- | --- | --- | --- | --- | --- | --- | --- | --- | --- | --- | --- | --- | --- | --- | --- | --- | --- | --- | --- | --- | --- | --- | --- | --- | --- | --- | --- | --- | --- | --- | --- | --- | --- | --- | --- | --- | --- | --- | --- | --- | --- | --- | --- | --- | --- | --- | --- | --- | --- | --- | --- | --- | --- | --- | --- | --- | --- | --- | --- | --- | --- | --- | --- | --- | --- | --- | --- | --- | --- | --- | --- | --- | --- | --- | --- | --- | --- | --- | --- | --- | --- | --- | --- | --- | --- | --- | --- | --- | --- | --- | --- | --- |

*bloq – below lower limit of quantification
